# Expert consensus–based clinical practice guidelines for Care and weaning procedures in tracheostomized ICU patients after invasive mechanical ventilation: a joint statement by the Intensive Care Physiotherapy Society (SKR) and the French Intensive Care Society (SRLF)

**DOI:** 10.1016/j.aicoj.2026.100045

**Published:** 2026-03-12

**Authors:** Clément Medrinal, Julie Delemazure, Manon Billard, Dorothée Carpentier, Gérald Choukroun, Yann Combret, Anne-Claire De Crouy, Carlos Díaz López, Jonathan Dugernier, Muriel Farcy, Adela Foudhaili, Thomas Gallice, Peggy Gatignol, Zina Ghelab, Marie-Hélène Houze, Mélissa Jezequel, Anne-Claire Latiers, Mehdi Marzouk, Elise Morawiec, Lise Piquilloud, Aude Ruttimann, Perrine Sanchez, Nicolas Terzi, Arnaud W. Thille, Matthieu Reffienna

**Affiliations:** a Univ Rouen Normandie, GRHVN UR 3830, Intensive Care Unit Department Le Havre Hospital, 76290 Montivilliers, Rouen University Hospital, Department of Pulmonary Rehabilitation, Rouen F76000, France; b AP-HP, Groupe Hospitalier Universitaire APHP-Sorbonne Université, Site Pitié-Salpêtrière, Service de Médecine Intensive et Réanimation (Département R3S), F-75013 Paris, France; c Service Médecine Intensive-Réanimation, Centre Hospitalier Albi, 81000 Albi, France; d Medical Intensive Care Unit, Rouen University Hospital, Rouen F76000, France; e Post-ICU Respiratory Weaning Unit, Forcilles Hospital, Ferolles-Attilly, France; f Physiotherapy Department, Le Havre Hospital, 76600, Le Havre, Erphan UR 20201, Paris-Saclay University, 78000, Versailles, France; g Département d’Anesthésie Réanimation, Service de Rééducation Post-Réanimation (SRPR), Hôpital Universitaire de Bicêtre, APHP, Université Paris-Saclay, Le Kremlin-Bicêtre, France; h Physiotherapy Department, Forcilles Hospital, F-77150, Férolles-Attilly, France; i Haute Ecole Arc Santé, HES-SO University of Applied Sciences and Arts Western, Delémont. Neuchâtel Hospital, Réseau Hospitalier Neuchâtelois, Neuchâtel, Switzerland; j AP-HP, Hôpital Raymond Poincaré et Hôpital Sainte Perine, Pharmacie, Unité Dispositifs Médicaux Stériles, 92380 Garches, France; k Université Paris Cité, Inserm U942 MASCOT, AP-HP, Département de Médecine Physique et Réadaptation, CHU Lariboisières, Paris, France; l CHU de Bordeaux, Hôpital Pellegrin, Réanimation Neurologique, Neurochirugie B, HDJ Déglutition, Equipe ACTIVE, BPH, INSERM U1219, Université de Bordeaux, France; m AP-HP, ENT Department, Pitié-Salpêtrière University Hospital, INSERM, UMRS1158 Neurophysiologie Respiratoire Expérimentale et Clinique, Sorbonne Université, Paris, France; n AP‐HP and University of Paris, Division of Paediatric Otolaryngology, Robert Debré Hospital, Paris, France; o AP-HP, CHU Lariboisière, Département de Médecine Physique et Réadaptation, F-75010 Paris, France; p Unité de Soins Intensifs de Cardiologie, Centre Hospitalier de Saint-Brieuc, Saint-Brieuc, France; q Service de Kinésithérapie et Ergothérapie, Cliniques Universitaires Saint-Luc, IRAII, Institut de Recherche Expérimentale et Clinique (IREC), Pôle de Pneumologie, O.R.L. et Dermatologie (LuNS, Lung-Nose-Skin), Université Catholique de Louvain (UCLouvain), Brussels, Belgium; r Médecine Intensive Réanimation, Centre Hospitalier D'Arras, Arras, France; s Department of Pneumology, Pitié-Salpêtrière Hospital, 75013 Paris, France; t Adult Intensive Care Unit, University Hospital of Lausanne and Lausanne University, Lausanne, Switzerland; u Service de Médecine Intensive-Réanimation, Hôpital Cochin, Assistance Publique - Hôpitaux de Paris, Paris, France; v Cabinet du Stiletto, Private Practice, Ajaccio, France; w Department of Medical Intensive Care, CHU Grenoble-Alpes, Grenoble, France; x CHU de Poitiers, Médecine Intensive Réanimation, Poitiers, France; y Hôpital Foch, Service de Réanimation Polyvalente, Suresnes, Université Paris Cité, Inserm, U1149 CRI, France Université Paris Cité, URP3625, Paris, France

## Introduction

Extubation failure after invasive mechanical ventilation occurs frequently. Several large-scale cohort studies have reported rates of failure between 14% and 18%, and 35% of patients had not been weaned at three months [[1], [2], [3]]. To facilitate weaning, tracheostomy is a procedure commonly used in ICU patients whose liberation from mechanical ventilation is unsuccessful. French expert guidance considers tracheostomy in adult ICUs as a procedure planned mainly as anticipation for prolonged weaning or after failed extubation; the panel suggests considering tracheostomy as an option when weaning extends >7 days after the first spontaneous breathing trial failure, and in acquired reversible neuromuscular disorders [4]. In 2018, French national insurance data on 77,132 critically ill patients indicated that 3,688 (4.8%) had undergone tracheostomy [5]. Tracheostomy rates reported in large cohorts ranged from 21% for all patients with separation attempts to 63% for patients in prolonged weaning [3].

Tracheostomy presents many advantages. Its purposes are well-known to clinicians: the ability to maintain mechanical ventilation and secure airway access, reduced work of breathing via decreased upper-airway resistance, improved patient comfort, decreased sedative exposure, liberation of the mouth to restore phonation and swallowing and promote mobilization. Nevertheless, these advantages should be weighed against uncommon but at times severe complications. Across contemporary cohorts, post-tracheostomy ventilator weaning typically takes about 12 days (IQR 7–20) in COVID-19 series, with 21% mortality and 93% of survivors weaned and 86% decannulated [6]. In a general ICU cohort tracheostomized for complex weaning, hospital mortality was 24%, 37.5% had a poor outcome (death or discharge with tracheostomy), 62.5% were alive and decannulated at hospital discharge, and the median time from intubation to decannulation was 42 days [7].

The management of tracheostomized patients is specific and requires adequate knowledge of the available devices, upper-airway physiology, and the mechanisms of phonation and swallowing. Many steps of weaning and bedside care remain insufficiently described, markedly heterogeneous, and largely practice-based. In light of these considerations, updated, standardized guidelines for post-tracheostomy ventilator weaning and decannulation in ICU patients are needed.

We are presenting updated, evidence-aligned guidelines for adult ICU tracheostomy care, from ventilator weaning through restoration of phonation and swallowing to decannulation. The guidelines are designed for the multidisciplinary teams managing tracheostomy across the ICU-to-ward continuum (intensivists, ICU physiotherapists, respiratory therapists, nurses, speech-language pathologists, otolaryngologists, thoracic surgeons, and rehabilitation professionals). They should also inform researchers and hospital leaders responsible for pathway design and policy, and serve as a teaching resource for trainees as well as for patients and families.

## Method

We defined the target population as adult patients with a tracheostomy performed during or following an ICU stay for prolonged or complex weaning, irrespective of timing (< or ≥ day 7) or technique (percutaneous or surgical). This definition was used both to guide our literature search (eligibility criteria) and to specify the population to whom our recommendations apply. Acquired, potentially reversible neurologic disorders were included (e.g., Guillain–Barré syndrome, brain or spinal injury and critical illness weakness).

The following were excluded: patients with burns; those tracheostomized for oncologic, otorhinolaryngology surgery, or traumatic upper–airway lesions; patients with progressive neuromuscular diseases (e.g., amyotrophic lateral sclerosis, myotonic disorders); and all paediatric patients.

The guidelines were developed by 25 experts in adult ICU care affiliated with the Intensive Care Physiotherapy Society (Société de Kinésithérapie de Réanimation, SKR) and the French Intensive Care Society (Société de Réanimation de Langue Française, SRLF). The experts used the Grading of Recommendations Assessment, Development and Evaluation (GRADE) methodology [8]. The steering committee (CM, JD, AWT and MR) identified key questions for the adult population in PICO format; these were validated by the expert task force and defined the scope of the literature search. For each question, the task force specified indexing terms, time limits, the target population, and prespecified outcomes. According to the GRADE, each retrieved study was assigned a level of evidence based on design and methodological quality. The panel then determined the overall level of evidence for each PICO question, considering the certainty across studies, consistency of results, and the balance of benefits and harms. A high overall level of evidence led to a strong recommendation (GRADE 1) (“should be done” or “should not be done”); a moderate or low level of evidence led to an optional recommendation (GRADE 2) (“should probably be done” or “should probably not be done”). In the absence of sufficient evidence, statements were issued as expert opinion; when evidence was contradictory or absent, the panel stated that no recommendation could be made.

Recommendations were discussed during five meetings of the full panel. Experts then rated each recommendation individually on a 1–9 scale (1–3, disagreement; 4–6, indecision; 7–9, agreement). A recommendation was considered approved if at least 50% agreed and no more than 20% disagreed. Strong agreement was defined as agreement by at least 70% of the experts. When strong agreement was not achieved, the recommendation was revised and re-rated. Only expert opinion statements having obtained strong agreement were adopted.

Results are presented as the formulated recommendation, followed by the strength of the recommendation, and the rationale that led to it. Where possible, a carbon footprint analysis was performed.

## Results

Across 24 PICO questions, the panel issued the following 25 assessments: no strong recommendation (GRADE 1), seven conditional recommendations (GRADE 2), ten expert opinion statements, and eight “no recommendations”.

R1: For tracheostomized (>72 h) adults patients undergoing ventilator weaning for respiratory or neurologic reasons, experts suggest performing daily stoma care without antiseptics.

### Expert opinion, strong agreement

To date, no study has compared povidone iodine versus no antiseptics for tracheostomy care on clinical outcomes (local infection, ventilator-associated pneumonia, skin lesions or comfort). A single randomized controlled trial [9] involving 100 patients compared polyhexanide versus saline + Povidone iodine for tracheostomy care, assessing the occurrence of local infections. No significant difference in local infection rates was observed between care with polyhexanide and care with Povidone iodine. In 2017, the CDC guideline recommended not to apply antimicrobial agents (ointments, solutions, or powders) to surgical incisions, the objective being to prevent surgical site infection [10]. These recommendations are consistent with those of the Haute Autorité de Santé, which specify that cleansing with mild soap or normal saline is sufficient to limit the risk of infection [11].

According to the Carebon app tool, the carbon footprint shows that use of antiseptics such as Betadine or chlorhexidine has a higher impact (e.g., 0.69 kgCO_2_e for 500 mL of Betadine vs 1.78 kgCO_2_e for 125 mL of chlorhexidine), whereas liquid soap or normal saline have a lower impact (about 0.47–1.29 kgCO_2_e per kilogram).

Accordingly, experts suggest not using antiseptics for standard daily stoma care, preferring mild soap, normal saline, or water.

R2: The experts suggest using foam dressings to limit the risk of pressure injury.

### Expert opinion, strong agreement

Hydrophilic polyurethane foam dressings are non-adhesive pads placed around the tracheostoma or beneath tracheostomy ties. They are designed to absorb secretions and maintain a moist peristomal environment; their typical thickness (∼0.5 mm) provides cushioning under the flange or ties and may help limit local pressure and friction [12]. They are analogous in composition and use to non-adhesive hydrocellular dressings for chronic wounds (e.g., leg ulcers). Evidence specific to tracheostomy care remains limited. To our knowledge, no studies published after the 2018 recommendations have provided additional data on the risk of local infection. Several studies on tracheostomy-related pressure injuries suggest lower incidence with foam-based dressings and associated preventive measures. In a meta-analysis, Moser et al. reported overall incidence of tracheostomy-related pressure injury of 17.0% in control groups versus 3.5% in intervention groups (an overall 79% relative reduction (95% CI, 63–96%) [13]. Because interventions in the active arms varied across studies, and since foam dressings were the common element among effective strategies, these findings cannot be attributed with certainty to a single component. Experts suggest applying a foam dressing around the stoma, ensuring it is not occlusive.

R3: Given the lack of data, no recommendation can be made regarding the type of oral care to be performed.

### No recommendation. Strong agreement

Oral care is routine in ICUs, with most of the evidence involving intubated patients; however, relevant findings are heterogeneous [4]. In a 2023 meta-analysis of four trials, povidone–iodine did not significantly reduce ventilator-associated pneumonia versus either placebo/standard oral care (pooled RR 0.61, 95% CI 0.25–1.47) or versus chlorhexidine (RR 1.50, 95% CI 0.46–4.87) [14]. In 2024, international guidelines from the Infectious Diseases Society recommended oropharyngeal care with tooth-brushing without chlorhexidine for patients ventilated in ICU via either an endotracheal tube or tracheostomy [15], due to potential adverse effects reported in some studies (increased mortality and infections) (16), of which the mechanisms remain to be clarified. In tracheostomized patients, one step-down (post-ICU) study compared ventilator-associated pneumonia rates under a standardized oral-care protocol (tooth-brushing +0.12% chlorhexidine every 12 h) with NHSN benchmark data for tracheostomy and endotracheal-tube cohorts [17]. No significant difference in ventilator-associated pneumonia incidence was found. However, the study had major limitations: non-comparability of groups (tracheostomized patients in the intervention vs a mixed intubated/tracheostomized population in the control) and a lack of information on the type and frequency of oropharyngeal care in the control group. Current ICU oral-care guidance supports tooth-brushing without routine antiseptics, given a lack of proven benefit and potential harms.

R4: Experts suggest not suturing tracheostomy cannulas outside of specific situations.

### Expert opinion, strong agreement

Fixation of the tracheostomy tube with neck ties alone or with additional skin sutures placed through the outer flange are two common techniques intended to secure the cannula and mitigate peristomal complications; skin sutures are used more often after surgical than following percutaneous tracheostomy. While accidental decannulation (unexpected tube removal from the stoma by the patient or during care) occurs infrequently in both early and late postoperative periods, it entails significant morbidity and mortality when it happens early after tube placement. In a multi-institutional retrospective analysis of 1,175 tracheostomies across eight academic centres, decannulation rates were <1% in both skin-sutured and non-sutured groups, with no significant difference between techniques [18]. A 2021 single-region retrospective study of 1,355 predominantly medical and trauma patients compared events within the first seven days after cannula placement and likewise found no statistically significant difference in accidental decannulation between Velcro neck ties and skin-sutured fixation (1.54% vs 1.11%); skin-suture fixation showed a numerically higher ulceration rate that was not statistically significant [19]. In addition to fixation technique, a prospective pre–post quality-improvement program focusing on staff education, risk identification, and equipment availability led to numerically reduced accidental decannulation (from 4.2 ± 0.9 to 2.7 ± 1.9 per 1,000 tracheostomy-days; p = 0.04) [20]. In conclusion, serious adverse events such as accidental decannulation are rare and not directly related to the fixation technique applied. Current observational data do not show lower dislodgement rates with skin-suture fixation compared with neck ties alone. Skin sutures should consequently be reserved for selected high-risk situations.

R5.1: For tracheostomized adults with an air-inflated cuff, experts suggest maintaining cuff pressure lower than 30 cmH_2_O to decrease the risk of tracheal injury.

### Expert opinion, strong agreement

R5.2: For tracheostomized adults with an air-inflated cuff, experts suggest maintaining cuff pressure greater than 20 cmH_2_O in order to limit the occurrence of ventilator-acquired pneumonia.

### Expert opinion, strong agreement

Evidence specific to tracheostomized adults on target cuff pressure is limited and imprecise, much of the rationale relies on studies in intubated patients, many of whom are elderly. Physiologically, elevated cuff pressures (>30 cmH_2_O) reduce tracheal mucosal capillary blood flow at the cuff–mucosa interface, predisposing to ischemia and subsequent scar-related injury leading to tracheal stenosis [21,22]. In this context, high-volume, low-pressure cuff designs were adopted to distribute surface pressure and mitigate mucosal injury, with clinical data (postoperative/anaesthesia settings) suggesting fewer cuff-related symptoms when pressures are controlled and high-volume, low-pressure cuffs are used [23,24].

Conversely, insufficient cuff pressure increases the risk of micro-aspiration of oropharyngeal secretions and gastric contents. In intubated patients, indirect evidence supports a threshold of ≥20 cmH_2_O to reduce micro-aspiration and potentially lower ventilator-associated pneumonia risk [[25], [26], [27]]; systems for continuous cuff-pressure control have been associated with decreased micro-aspiration and/or ventilator-associated pneumonia in observational or interventional studies, albeit with moderate-to-low certainty [26,27].

Overall, this extrapolated literature aligns with manufacturer guidance recommending a 20–30 cmH_2_O range: below 20 increases aspiration risk, above 30 increases mucosal injury risk.

R6: Given the lack of data on clinical outcome, no recommendation can be made regarding the use of tracheostomy cannulas with or without an inner cannula.

### No recommendation. Strong agreement

Practices for the use and replacement of inner cannulas vary widely and rely more on clinical experience than on robust comparative data. In a pilot ICU study, Burns did not show that frequent inner-cannula changes reduced obstruction or colonization, thereby suggesting that the routine may be superfluous, albeit with the caveat that systematic suctioning was performed in the study cohort [28]. Microbiologically, observational series have documented near-constant biofilm on tracheostomy tubes in both inpatient and ambulatory settings; moreover, a largely outpatient study reported an inverse correlation between the frequency of inner-cannula changes and colonization events, a signal insufficient to establish causality [29]. Experimental data consistently show that inserting an inner cannula reduces lumen section diameter and increases resistive load: on bench testing, placement of the inner cannula increased inspiratory/expiratory resistance by roughly two- to threefold, while removal significantly decreased imposed ventilatory work [[30], [31], [32]]. Other tests have confirmed that tube and inner-cannula type (model, size) alter pressure–flow curves and inspiratory/expiratory resistance, with substantial variability across devices [30,32]. Secretions within the inner cannula represent a major risk for obstruction [33]; however, removal of the inner cannula can relieve obstruction when suction alone is insufficient [30]; this can be considered as an advantage. In conclusion, the available evidence does not support recommending for or against systematic inner-cannula use; decisions should remain individualized according to secretion burden, monitoring capacity, and weaning dynamics [[28], [29], [30]].

R7: Normal saline instillation should probably not be performed routinely during tracheal suctioning.

### Grade 2−. Strong agreement

Normal saline instillation before endotracheal suctioning remains common, due to a strong perceived benefit among nurses despite limited supporting evidence [34,35]. Current literature, however, questions the effectiveness and safety of routine use. A 2017 meta-analysis pooling five RCTs (n = 337) reported higher volumes recorded as “secretions” after instillation; since the recovered volume includes the instilled saline, this endpoint does not demonstrate increased mucus yield [36]. In another systematic review, only one out of two studies showed an increase; moreover, not all saline is retrieved by suctioning, with gravity-dependent pooling of residual fluid often described in the posterior right lower lobe [35]. Regarding airway obstruction, comparative data are sparse: a randomized trial from 2009 (n = 262) found no difference in endotracheal tube occlusion between instillation and control [37]. The frequently cited notion that instillation “stimulates cough” remains anecdotal in this context [35,37]. Desaturation typically follows instillation, with larger volumes producing greater declines and slower return to baseline; in tracheostomized patients with pneumonia (n = 16), testing 0, 2, and 5 mL boluses produced dose-related desaturation, with recovery exceeding five minutes after 5 mL [38]. At the meta-analytic level, SpO₂ at five minutes was lower with instillation (pooled mean difference: −1.14%) [36], and more recent synthesis likewise concludes that harms tend to outweigh benefits [39]. The effects on pneumonia remain unsettled. The same 2009 RCT reported less microbiologically proven VAP with instillation (23.5% vs 10.8%) (p = 0.008), yet earlier mechanistic work cautions that saline plus suction may displace bacteria into the lower airways [34,37]. Given the real-world prevalence of the practice (88% of ICU nurses report using it) and the balance of current data (desaturation without clear airway-clearance benefit and conflicting infection signals) routine saline instillation should be avoided in tracheostomized adults; if nevertheless considered, it should be selective and justified by patient-specific factors.

R8: Mechanical ventilation weaning should probably use off-ventilator breathing sessions rather than by progressively reducing ventilatory assistance.

### Grade 2+. Strong agreement

Weaning by staged spontaneous-breathing sessions disconnected from the ventilator is supported by a randomized trial in 316 adults undergoing prolonged weaning who were transferred to a long-term acute care hospital [40]. In this one study on the topic, consecutive tracheostomized patients ventilated for at least 21 days were screened; exclusion criteria included cardiopulmonary instability, profound neurologic deficits, bilateral phrenic-nerve injury, prior admission, and limited life expectancy. Out of 500 screened patients, 160 (32%) immediately tolerated 120 h (5 days) of unassisted tracheostomy-collar breathing without respiratory distress and were consequently not randomized (roughly one out of three transferred patients remained on mechanical ventilation despite being immediately ready to wean). This highlights the value of a structured screening process to verify spontaneous breathing ability and to attempt prompt ventilator disconnection. This also suggests that weaning potential may be under-recognized in daily practice.

Patients who developed respiratory distress during the 5-day screening window were randomized to subsequent staged tracheostomy-collar disconnections or to progressive pressure-support (PS) reduction. For randomization, eligible subjects were stratified by time to failure (early failure, 0–12 h; late failure, 12–120 h). In the tracheostomy-collar arm, disconnections followed a strict schedule: up to 12 h on days 1–2 with overnight reconnection, up to 24 h per day from day 3 onward. In the PS arm, tolerance to lower support was assessed three times daily; PS was initially set to target a respiratory rate <30 breaths/min, then decreased in 2 cmH_2_O steps up to three times per day. When PS was reduced to 6 cmH_2_O with sustained tolerance, patients proceeded to disconnection.

In the randomized cohort, staged disconnections shortened weaning time (15 [8–25] vs 19 [12–31] days; p = 0.004) without differences in 6- or 12-month mortality. Of note, staged spontaneous-breathing sessions off the ventilator outperformed pressure support in the subgroup that failed between 12 and 120 h, but not among those who failed within 0–12 h. Taken together, and given the operational simplicity of staged disconnections, progressive off-ventilator sessions appear to be a reasonable default approach for tracheostomized adults in the later phase of prolonged/complex weaning, at least for those able to tolerate more than 12 h of spontaneous breathing after the first disconnection attempt. This strategy naturally coheres with our other recommendations (see R9 and R10 on cuff deflation and speaking-valve use during tolerated sessions).

R9: Periods of ventilator liberation should probably be performed with the cuff deflated.

### Grade 2 + . Strong agreement

One randomized controlled trial evaluated whether increasing the effective airway diameter, by deflating the cuff and using a fenestrated tracheostomy cannula with a 7-mm inner diameter during ventilator disconnections affected outcomes in 181 tracheostomized adults (deflated, n = 94; inflated, n = 87). In the control group, an 8-mm inner-diameter cannula with an inner sleeve was used with the cuff inflated [41]. Patients with severe brain injury (Glascow score <6/15) and with severe swallowing disorders evaluated with a 50 ml water drink test with cuff deflated were excluded. Crucially, all patients first passed a tube-occlusion test: with the cuff deflated, the tracheostomy was briefly occluded so that airflow traversed the upper airways to confirm supraglottic patency. During disconnections, both groups received heated, humidified high-flow oxygen via the tracheostomy; the deflated group received higher flow (69 vs. 55 L/min). As regards keeping the cuff inflated, deflation was associated with earlier ventilator liberation (median 3 [[2], [3], [4]] vs 8 [[6], [7], [8], [9], [10]] days to successful 24-h disconnection) and fewer respiratory infections (ventilator-associated pneumonia or tracheobronchitis 20% vs 36%) [41]. Mechanistically, deflation increases the effective airway diameter and may support swallowing by restoring subglottic pressure and upper-airway airflow. Swallowing studies are broadly consistent with this signal: in a crossover series (n = 12), feeding with an inflated cuff predicted aspiration, with a non-significant three-fold reduction once the cuff was deflated (7% vs 18%) [42]; physiological work shows shorter swallow-reflex latency, greater laryngeal elevation, and increased anterior hyoid motion when the cuff is deflated [[43], [44], [45]]. A retrospective comparison of video-fluoroscopic swallow exams reported less silent aspiration with the 281 patients with deflated cuff (7%) vs 23% for the 342 patients with inflated cuff) [46]. In a randomized video-fluoroscopic crossover study on swallow physiology (n = 18), cuff deflation alone did not change penetration/aspiration across bolus consistencies, but the penetration–aspiration score for liquids was halved when a one-way valve was added to a deflated cuffed tube, consistent with the benefits from routing airflow through the upper airway [45]. Pragmatically, cuff deflation accelerates weaning from ventilator and protects from respiratory infection; moreover, it permits canula occlusion (one-way valve or capping); the step that actively redirects airflow through the upper airway is detailed separately in Recommendation 10.

R10: The experts suggest using a speaking valve, with the cuff deflated, during periods of ventilator liberation.

### Expert opinion, strong agreement

This expert opinion is based on 11 studies (n = 149). Several provide indirect evidence (neuromuscular or spinal cord–injury populations, mixed cohorts of ventilated and non-ventilated patients, or bench studies) (47–52). Only one randomized controlled crossover single blinded study was identified (n = 18) [52]. No trial has evaluated mortality, decannulation, or ICU/hospital length of stay.

A speaking valve can only be used with the cuff deflated. With the valve in place, air may be inspired through the valve and canula, as well as through the upper airways around the canula. During expiration, the flap closes and flow is redirected to the upper airways, enabling phonation.

Use of a speaking valve may improve swallowing, particularly by reducing the number and severity of aspiration events [45,[53], [54], [55]]. This effect could be explained by the restoration of expiratory flow toward the upper airways after swallowing [48]. The valve does not appear to affect ventilatory–swallowing coordination, the number of swallows per minute, or the ability to take oral nutrition [48,50,51]. Other data suggest potential benefits for mobility, olfaction, and pulmonary recruitment [52,55,56]. Importantly, the valve does not significantly increase the work of breathing (no accessory-muscle recruitment and or increase in measured work of breathing) and effectively redistributes inspiratory flow toward the upper airways [47,49,50]. No adverse events were reported, nor were meaningful changes observed in respiratory rate, heart rate, PaCO₂, or SpO₂ at the time of valve placement. Finally, because part of inspiratory airflow shunts the upper airways when the speaking valve is used during a long period, particularly when supplemental oxygen is delivered via the tracheostomy, it may predispose to drying [49]; clinicians should monitor mucosal dryness and secretion viscosity and adjust humidification accordingly during extended sessions.

R11: The experts suggest that tracheostomy cannula changes should not be performed systematically.

### Expert opinion, strong agreement

Many tracheostomy tubes are certified with short-term intended use (≤30 days). As a result, manufacturers’ IFUs (instructions for use) commonly limit continuous use to approximately 28–30 days [57]. The 2012 UK consensus conference suggest that the timing window for the first tube change is between day 10 and day 14 after percutaneous dilatational tracheostomy [58]. In ICU practice, French recommendations emphasize individualizing to the patient and point out that early changes carry risks (dislodgement, respiratory arrest) (4). Observational data on the optimal timing of the first tube change after surgical or percutaneous tracheostomy indicate that performing the first change before day 7 shortens both ICU and hospital length of stay without increasing complications [59]. This initial change often involves downsizing. A Korean retrospective study trying to determine the optimal timing of the first change allocated approximately 4,000 patients into three groups (≤6 days; 7–9 days; ≥10 days); in that study, all-cause mortality was lowest in the 7–9 day group, and the risk of pulmonary complications increased when the tube was changed within the first six days [60]. Other limited retrospective data suggest that instituting a 15-day routine tube-change procedure may reduce the incidence of granulomas [61].

In the absence of evidence supporting calendar-based replacement, and consistent with our prior recommendation, we do not recommend routine tracheostomy tube changes.

R12: A tracheostomy tube change should probably be integrated into the weaning strategy and considered when the aim is to increase effective airway space (e.g., by downsizing or switching tube type).

### Grade 2+. Strong agreement

After confirming upper-airway patency with the tube-occlusion test, a trajectory consisting of off-ventilator sessions (R8), cuff deflation (R9) and speaking-valve use (R10) often allows progress without any additional intervention on the tube. In a long-term acute care hospital (LTACH) for difficult weaning, approximately 32% of patients were liberated with no intervention other than transfer to the LTACH [40]. However in a manometry-guided cohort, speaking-valve use was feasible in 78% with the initial tube, increasing to 95% after individualized optimization [62]. When occlusion or speaking-valve use remains poorly tolerated, whether clinically or on tracheal manometry, targeted tube optimization to increase effective airway space around the tube becomes appropriate, most often through downsizing, as described in Hernández et al. [41]. Clinically, after individualized adjustment, pressures fall, and tolerance of speaking-valve or capping widens, and decannulation is facilitated [62]. Observational data also associate downsizing with successful decannulation (adjusted OR = 6.5) [63]. Taken together, these findings support adoption of a needs-based rather than a routine optimization strategy. It would consist primarily in downsizing and, where appropriate, a type change that reduces intraluminal burden, when the R8–R9–R10 steps are poorly tolerated, consistent with R11 (no systematic routine tube changes).

R13: No recommendation can be made regarding a high-flow therapy device during periods of ventilator liberation.

### No recommendation. Strong agreement

In tracheostomized adults disconnected from the ventilator, the evidence base for high-flow therapy delivered through the tracheostomy (HFT) is short, physiologic, and discordant. A randomized crossover comparison reported a small, rapid rise in oxygenation with HFT, with higher SpO₂/FiO₂ at 5 and 15 min than with a T-piece, without meaningful differences in airway pressure, end-tidal CO₂, or respiratory rate, suggesting only a modest immediate oxygenation benefit [64]. By contrast, two physiologic crossover studies found no measurable advantage of HFT over T-piece on load and drive [65,66]. Using electrical activity of the diaphragm, neuro-ventilatory drive, Pressure Time Product muscle, respiratory rate, PaCO₂, and gas exchange were unchanged across HFT and T-piece periods after disconnection, and oxygen targets were achieved at similar FiO₂, making a relevant positive-pressure effect unlikely [65]. In a separate randomized crossover study, inspiratory effort measurement determined by simplified oesophageal pressure–time product and breathing frequency increased when patients moved from PSV to unassisted breathing, but did not differ between HFT and T-piece; in contrast; oxygenation was higher with T-piece compared to HFT (p = 0.02) [66]. Taken together, these physiologic data indicate no consistent reduction in work of breathing or CO₂ with HFT when compared with a T-piece during deventilation. These results were consistent with a bench study that evaluated the work of breathing across tracheostomy modalities, including HFT [49].

One possible explanation is that, unlike high-flow nasal therapy, which reduces neuro-ventilatory drive through nasopharyngeal dead-space washout, with a modest PEEP effect, tracheal delivery bypasses the upper-airway reservoir, in such a way that CO₂-clearing and resistance-lowering mechanisms are largely absent. Small oxygenation gains with HFT have been observed at higher tracheal flows around 50 L/min, but they remain modest and are not consistently accompanied by improvements in PaCO₂ or work of breathing [67].

Overall, current evidence does not support HFT within a standard deventilation pathway for tracheostomized adults. HFT can be considered selectively when oxygenation is low or requires high FiO₂ and humidification, but there are no robust data showing advantages on weaning duration, decannulation, or mortality.

R14: No recommendation can be made regarding active versus passive humidification during periods of ventilator liberation.

### No recommendation. Strong agreement

Heating and humidifying inspired gas through the tracheostomy tube in the spontaneously breathing period during ventilator liberation is essential once the upper airways are bypassed. Adequate conditioning preserves mucociliary function and epithelial integrity and helps prevent tube or airway occlusion by secretion plugs. In this setting, humidification can be delivered with a heat-and-moisture exchanger combined with a microbiological filter (HMEF) or with a heated humidifier directly connected to the tracheostomy (High-Flow Therapy, HFT) [68]. Even though external “cool-mist” systems are outside the scope of these recommendations (they are evaluated only in post-laryngectomy care), it should be noted that in several populations, such devices were associated with more mucus plugging, pulmonary complaints (cough, expectoration), higher care needs, poorer sleep, and reduced mobility compared with HMEFs [[69], [70], [71]].

Choosing between HMEFs and HFT during periods of ventilator liberation cannot be determined on the basis of the limited evidence available. In a cross-over study of 10 stable tracheostomized subjects (supplemental oxygen ≤1 L/min), Nakanishi et al. reported adequate absolute humidity with both techniques (>28 mg H_2_O/L), with higher absolute humidity under HFT (≈40 mg H_2_O/L) than with an HMEF (≈30 mg H_2_O/L) [72]. However, no clinical outcome advantage has been demonstrated for the higher humidity achieved with HFT.

Clinicians should also be aware of device-to-device variability among HMEFs. Two bench studies designed for spontaneously breathing tracheostomized patients found that several HMEFs failed to deliver ≥28 mg H_2_O/L under typical conditions, and that performance deteriorated with cold, dry oxygen supplementation and low-minute ventilation [73,74]. Because HMEF performance is affected by dry oxygen, HFT is increasingly considered for hypoxemic tracheostomized patients, but this practice remains assumption-based (improved mucociliary clearance with warmer, wetter gas) rather than evidence-based. A preliminary study in lung-transplant recipients (n = 27) suggested a higher decannulation rate at ICU discharge with HFT up to 60 L/min versus oxygen via HMEF (93% vs 46%, p = 0.01), and good tolerance of tracheal HFT, but the small, nonrandomized design and specific case-mix limit generalizability [75].

Adequately powered trials are needed to compare these gas-conditioning strategies in spontaneously breathing tracheostomized adults, with outcomes that matter clinically: progress of ventilator weaning and readiness for decannulation; patient-reported comfort, dyspnoea, sputum clearance, and sleep quality; incidence of tube occlusion; gas exchange and work of breathing; nursing workload (suctioning, early mobilization); and, importantly, cost and environmental impact.

R15: There is no evidence to recommend application of a standardized ventilator-liberation protocol.

### No recommendation. Strong agreement

Most of the evidence on weaning protocols comes from intubated, sedated patients; its applicability to tracheostomized adults in the weaning phase, who are more awake and for whom restoration of laryngeal airflow, phonation, and swallowing are central, is limited [76]. Moreover, the data specific to tracheostomized patients are too heterogeneous to support or refute a standardized ventilator-liberation protocol. The only randomized trial similar to a “protocolized” strategy evaluated discrete components (cuff deflation and tube downsizing) within an otherwise identically structured program in both arms; it essentially tested increasing effective airway space around the tube, rather than “protocol versus no protocol” [41]. Physiologic studies conducted during disconnection report modest or neutral effects of oxygen-delivery modalities on ventilatory load and respiratory drive, underscoring variability by patient phenotype and timing in the weaning trajectory [[77], [78], [79], [80], [81]]. Accordingly, current evidence does not support issuing a recommendation for a standardized ventilator-liberation protocol in tracheostomized adults. Certain components can nevertheless be standardized locally (e.g.: daily assessment, spontaneous breathing sessions, cuff deflation, occlusion trials with a one-way speaking valve). In this context, standardized protocols can be implemented as part of local quality-improvement initiatives; a recent single-centre quality-improvement initiative implemented a standardized tracheostomy weaning protocol and reported a reduction in the median duration of mechanical ventilation after tracheostomy (from 17 to 10.6 days), along with a shorter ICU length of stay (−4.3 days) [82]. Future studies should explicitly compare with versus without protocol, using robust outcomes such as post-tracheostomy ventilation duration, complications, ICU and hospital length of stay, decannulation rate, and mortality.

R16: For tracheostomized patients tolerating a phase of off-ventilator breathing, threshold-loading inspiratory-muscle training should probably not be used.

### Grade 2−. Strong agreement

Five randomized controlled trials (RCTs) (n = 385) and one prospective study (n = 10) were identified [[83], [84], [85], [86], [87], [88]]. The Inspiratory Muscle Training (IMT) programs used a fixed- or variable-threshold valve attached to the tracheostomy, generating inspiratory load when the patient was off the ventilator. No study evaluated changes to ventilator trigger settings. IMT was delivered 1–2 times/day, 5–7 days/week, for 30–60 repetitions per session. Resistance was fixed or adjustable, individualized from Pi_max_ (about 30% at initiation), and re-titrated from daily to weekly. All patients underwent daily off-ventilator sessions in both groups aimed at determining the longest tolerated duration with predefined stopping criteria, which could be considered as a kind of IMT.

Across studies, results were discordant for weaning success (4 RCTs, n = 364) and mortality (2 RCTs, n = 191) [83,84,86,87]. There was no change in weaning duration (3 RCTs, n = 236) [83,84,86]. IMT had no impact on total duration of mechanical ventilation (4 RCTs, n = 284) or on ICU length of stay (2 RCTs, n = 194) [83,84,86,87]. Most trials did not show increased inspiratory-muscle strength with IMT (5 RCTs, n = 385) [83,84,86,87]. If its efficiency remains unproven, safety seems to be uncompromised, insofar as only mild, transient destabilizations, chiefly respiratory, were reported during IMT sessions; no serious adverse events occurred [83,86,88].

It should be noted that a single RCT with 104 patients reported positive findings across all evaluated outcomes, but the risk of bias was moderate due to the randomization process. Baseline imbalance in arousal level (62% vs 30% awake in favour of the IMT arm) could have influenced the conduct of off-ventilator sessions to the disadvantage of the control group [84]. Overall, compared with daily maximally tolerated off-ventilator sessions, IMT does not provide benefits.

R17: There is not enough data on clinical outcome to recommend instrumental cough-assistance techniques.

### No recommendation. Strong agreement

Because of the limited data available in tracheotomized patients undergoing weaning in an intensive care setting, no specific recommendations can be issued. However, insights may be extrapolated from studies on extubation in ICU and on patients with neuromuscular disorders not involved in a weaning process.

When drawing a parallel between decannulation failures and extubation failure, identifying patients who may require respiratory or cough assistance remains challenging [89,90]. Critically ill patients receiving mechanical ventilation via endotracheal intubation often have impaired airway clearance during intubation and after extubation because of ineffective cough and respiratory-muscle weakness or paralysis related to ICU-acquired weakness [[91], [92], [93]]. Excess respiratory secretions are a primary cause of extubation failure in ICUs and can delay extubation [94]. In routine practice outside the ICU context in patients with neuromuscular disease with or without tracheostomy, different kinds of airway clearance techniques such as mechanical insufflation–exsufflation (MI-E) are commonly initiated when peak cough flow (PCF) is <270 L/min [95]. MI-E delivers positive-pressure insufflation immediately followed by negative pressure to generate rapid expiratory flow and assist sputum clearance [95]. In a Cochrane review including intubated and tracheostomised patients in ICUs, very low-quality evidence suggested that cough-augmentation techniques might shorten the duration of mechanical ventilation without increasing harm, but overall evidence was insufficient to determine effects on weaning success, ventilation and weaning duration, tracheostomy and decannulation rates, length of stay, or mortality [96]. A recent scoping review in invasively ventilated critically ill adults (28 studies, only four including tracheostomized patients) highlighted marked heterogeneity in how MI-E is applied and reported, limiting the feasibility of any best-practice recommendations [97].

R18: No recommendation can be made on trans-laryngeal airflow techniques to restore phonation in patients who remain mechanically ventilated with the cuff inflated.

### No recommendation. Strong agreement

This recommendation applies to interventions that restore upper-airway airflow through the larynx while patients remain invasively ventilated: (i) in-line one-way speaking valves used with the cuff deflated and appropriate ventilator adjustments; (ii) above-cuff vocalization (ACV), (iii) leaked ventilation with cuff deflated. Across studies, evidence is dominated by feasibility/physiology with heterogeneous protocols and few comparative clinical endpoints, as underscored by a multinational qualitative study highlighting wide practice variation, implementation uncertainty, and the need for standardized ACV procedures and competencies [98].

In-line one-way speaking valves are used during mechanical ventilation with cuff deflated. An Australian cardiothoracic ICU series reported improved communication without prolonging ventilation time and showed signals of improved ventilation distribution and recruitment on electrical impedance tomography in 20 patients [52]. A systematic review concluded that most published work centres on feasibility, tolerance, and physiology, with limited controlled data on patient-important outcomes [99].

ACV is the only way to produce speaking with the cuff inflated by adding an adjusted 1−4 l/min gas flow on the above cuff line made for secretion aspiration. Gas flow is directed through the upper airway, across the vocal cords, to allow phonation. Early descriptions and a service evaluation demonstrated technical feasibility and safety when delivered by trained teams under protocolized conditions [100,101]. A hospital-wide pre–post implementation associated ACV with earlier return of speech and no major complications, while not proving causality [102]. In ventilated patients unable to tolerate cuff deflation, a randomized controlled trial of a talking tracheostomy tube improved voice-related quality of life, again without effects on weaning/decannulation endpoints [103]. Overall, across modalities, the literature is heterogeneous, mainly single-centre, and primarily physiologic/implementation-focused; robust comparative data on weaning duration, decannulation, ICU/hospital length of stay, or infection remain insufficient and inconsistent [99,104].

Phonation with cuff deflation and ventilator adjustments (usually volume assist–control with increased Vt/PEEP and longer Ti) may be feasible in chronic neuromuscular disease (e.g., high cervical spinal cord injury), in which airway protection is preserved and sufficient tidal volume can be delivered despite cuff deflation. Speech often occurs during inspiration. Use is uncommon and evidence is limited. Consequently, no recommendation can be issued for routine use during mechanical ventilation.

R19: No recommendation can be made regarding the use of fenestrated tracheostomy tubes.

### No recommendation. Strong agreement

Fenestrated tracheostomy tubes incorporate an opening in the outer curve of the cannula that permits expiratory flow to be redirected toward the larynx and thereby enables phonation. The fenestrated inner canula is usually coupled with cuff deflation so that gas can reach the upper airway.

The clinical literature suggests that a cannula with fenestration can help to restore voice in selected patients, but with a low level of evidence. In a 10-year retrospective series, 2000 tracheostomised patients were evaluated, but only 15 had a fenestrated cannula: 13/15 patients received a fenestrated tube for phonation issues and 12/13 achieved audible speech; 9 (60%) patients developed complications, most likely granulation [105]. A contemporary synthesis of ICU communication devices, covering in-line or tracheostomy speaking valves, and tube designs (fenestrated tube, cuff tight-to-shaft…) confirms that multiple routes to voice are available and that secretion burden and upper-airway patency are major determinants of audibility and clarity rather than the device alone [106]. Effects on resource use are mixed: across observational reports and small case series (often n < 20), there is no consistent indication of shorter ICU/hospital stays or faster decannulation, with outcomes largely reflecting case mix and severity [105,106].

Safety concerns are prominent and must be weighed carefully. In the Pandian cohort, 9/15 (60%) developed complications, granulation alone or in combination with tracheomalacia and/or tracheal stenosis [105]. In a large secondary analysis of hospital records (6,400 consultations, 4,284 adults), fenestrated tubes were rare (n = 17) but they were significantly associated with tracheostomy-related pressure injury [107]. Mechanistically, malalignment of the window [32], sharp edges at the fenestration, secretion crusting, and cuff interactions can contribute to mucosal injury, reinforcing the need for meticulous selection, sizing, humidification, and surveillance.

Fenestrated tubes are described as a potential option to help restore voice and promote one-way speaking valves or capping; however, the evidence base is limited to small, single-centre series, with no comparative data demonstrating earlier phonation, shorter length of stay, or faster decannulation versus other strategies [[105], [106], [107]]. In two studies, safety concerns connected with fenestrated tubes are non-trivial, with high rates of granulation and airway injury reported and an association with tracheostomy-related pressure injury [105,107].

R20: The experts suggest initiating swallowing management early, rather than delaying while awaiting specialist input.

### Expert opinion, strong agreement

Among ICU subgroups, tracheostomized patients are at particularly high risk of dysphagia [[108], [109], [110], [111]]. Prevalence may reach 93% [113]. Decannulation may fail in up to 41% of tracheostomized patients undergoing prolonged weaning, with severe dysphagia identified as the primary cause in 64% of cases [112].

Despite recognition of these risks, dysphagia management is often delayed and not well-codified. International surveys highlight major organizational gaps: only 4%–20% of ICUs have access to dedicated dysphagia specialists, and formalized protocols are lacking in most centres [[113], [114], [115]]. In a retrospective study on tracheostomised patients, some of whom were in ICUs, speech-language therapy began a median of 14 days after tracheostomy, with oral-intake decisions occurring accordingly late: oral intake took place 15 days after tracheostomy [116].

While prospective data specific to tracheostomized populations are lacking, indirect evidence supports early intervention. In post-extubation dysphagia, each day of delayed therapy increases the risk of persistent dysphagia or in-hospital mortality (adjusted OR 1.09; 95% CI, 1.02–1.18; p = 0.009), and is associated with dysphagia or death at days 7, 14, and 28 [117]. In a retrospective study about non-tracheostomised patients with stroke, delays >24 h in dysphagia assessment were associated with a 3% absolute increase in aspiration pneumonia; the most belatedly assessed patients had nearly twice the risk (adjusted OR 1.98; 95% CI, 1.67–2.35) [118].

Although high-quality evidence on the specific benefit of early specialist involvement in critical tracheostomized patients is lacking and even if studies of multidisciplinary approaches remain heterogeneous, the rationale for early screening is supported by the postulated risk of delaying and questions on implementation feasibility. Several bedside tools have been validated to detect aspiration in critical-care settings and are used by trained ICU clinicians (nurses, physicians, allied health) ([119–,120,121]). In the absence of broad specialist availability, early screening and initial management by trained ICU staff represents a clinically grounded and operationally realistic response to a well-documented high-risk condition. This approach prioritizes patient safety through timely risk identification while optimizing available specialized resources through appropriate triage and referral pathways.

R21: The experts suggest that gastrostomy should not be systematic but should be considered in light of nutritional status and swallowing assessment.

### Expert opinion, strong agreement

In adults with dysphagia, gastrostomy is typically considered when enteral feeding is expected to last >4 weeks. A meta-analysis shows fewer intervention failures than with a nasogastric tube (dislodgement/occlusion), better adherence and quality of life, and a more favourable course of nutritional markers (albumin, mid-arm circumference) ([122]). The ESPEN 2018 and ASPEN 2021 ICU nutrition guidelines do not make a specific recommendation regarding gastrostomy placement in adult intensive care, reflecting an insufficient level of evidence in this setting [123,124]. In neurology, ESPEN suggests early percutaneous endoscopic gastrotomy (PEG) in stroke patients ventilated for >48 h (consensus 85%), a recommendation that targets a neurological subgroup rather than the post-tracheostomy weaning pathway [123]. In neurocritical cohorts where tracheostomy and gastrostomy are often paired, several retrospective series evaluating early vs late PEG suggest shorter ICU and hospital stays with the early strategy, but interpretation is limited by severity bias and health-system organization (North American models), with no consistent signal on duration of ventilation, decannulation, pneumonia, or mortality [[125], [126], [127], [128]].

The relevant randomized controlled trials are not specific to tracheostomized patients: in ventilated stroke/traumatic brain injury intubated patients, very early PEG reduced the incidence of ventilator-associated pneumonia from 38% to 10% over three weeks, without an effect on mortality [129]. Two small-scale RCTs on mixed population, gastroesophageal reflux during mechanical ventilation through orotracheal tube report discordant results: negative [130] versus positive in a highly selected cohort with proven reflux and recurrent ventilator-associated pneumonia [131]. The hypothesis that early removal of the nasogastric tube might be beneficial (less iatrogenic dysphagia) is plausible when post-ICU dysphagia and its consequences are taken into account, but it has not been demonstrated specifically in tracheostomized patients undergoing weaning.

In tracheostomized adults undergoing weaning, the available evidence does not justify routine gastrostomy at the time of tracheostomy. The decision should be individualized by assessment of swallowing and aspiration risk, nutritional status, anticipated duration of non-oral feeding and weaning trajectory.

R22: Endoscopy before decannulation should probably not be systematically performed.

### Grade 2−. Strong agreement

Fibreoptic endoscopic evaluation of swallowing (FEES) is a versatile, high-yield tool because it directly characterizes dysphagia and upper-airway obstacles relevant to decannulation. When combined with clinical assessments (capping/occlusion trials, level of arousal and tone, cough), studies report very good sensitivity/specificity for predicting decannulation success [132]. There are nonetheless two reasons not to place it first-line: first, in most series described up until now, the decannulation decision was contingent on the FEES result, making it impossible to compare alternative assessments on complications or failure; second, several authors argue that the current “gold standard” thresholds may be overly conservative for swallowing [133,134]. When instrumental evaluation is unavailable, structured clinical assessment and dye tests remain useful and show positive correlation with FEES despite heterogeneous protocols [135]. The blue-dye test, however, has debatable sensitivity; it should be performed with the cuff deflated and the tracheostomy occluded (finger, one-way valve, cap) and repeated so as to reduce false negatives [136,137].

Most studies combine FEES with clinical evaluation (capping tolerance, arousal/tonus, cough) to guide decannulation. Laryngo-tracheal lesions, of which the incidence increases with tracheostomy duration, weigh on decannulation prognosis; FEES is valuable as a means of identifying them and directing management (medical, surgical, or cannula adjustment). However, these lesions are often pauci-symptomatic and, in and of themselves, do not preclude successful decannulation [[138], [139], [140], [141]]. Several cohorts have consistently proposed decannulation protocols without routine FEES, using occlusion/capping to unmask persistent upper-airway obstruction or dysphagia; reported failure rates are low, with FEES reserved for intolerance to capping or unexpected setbacks [[142], [143], [144]].

In ICU settings, transient dysphagia is frequent [113]. Pragmatically, FEES should be positioned as second-line, when occlusion/capping is not tolerated or when dysphagia fails to improve despite appropriate therapy; clinical screening and capping nonetheless remain the first step. Randomized studies are needed to compare clinical-first versus instrument-first strategies on patient-important outcomes.

R23: For tracheostomized patients weaned from mechanical ventilation, without major swallowing disorder and after verifying upper-airway patency, the decision to decannulate should probably be guided by the frequency of airway suctioning rather than conditioned on the success of a standardized 24 -h capping trial

### Grade 2+. Strong agreement

Many protocols condition decannulation on a patient’s ability to tolerate complete tracheostomy occlusion, with widely varying target durations [145]. The theoretical rationale is threefold: (1) to verify upper-airway patency; (2) to re-afferent the upper airways and thereby promote restoration of swallowing; and (3) to confirm the ability to breathe through the suprastomal dead space [48,50,99]. However, a speaking valve or a cap can increase ventilatory load: suprastomal dead space alone accounts for ∼30% additional work of breathing [146], compounded by resistance from the reduced tracheal lumen created by the tube itself. In frail patients, this additional resistive load can contribute to decannulation failures or prolonged weaning.

A prospective ICU randomized controlled trial included 330 tracheostomised patients weaned from mechanical ventilation [147]. Patients should successfully tolerate a 5-minute tracheostomy occlusion (capping) trial and have no severe swallowing dysfunction on a 50-mL water swallow test; both assessments are performed with the cuff deflated. In both arms, the cuff was deflated and oxygen/humidification were delivered via a tracheal interface with high flow therapy at 60 l/min. Both groups first underwent a brief capping/occlusion tolerance test to screen for upper-airway obstruction. Participants were then randomized to (i) a suction-frequency criterion for decannulation (≤2 suctions per 8-h block over 24 h) without requiring 24 h of continuous capping, or (ii) a protocol requiring 24 h of continuous capping. In this group, capping also started on a suction-frequency criterion. The suction-frequency protocol achieved shorter weaning time (6 [5–7] vs 13 [11–14] days; absolute difference: 7 days, 95% CI 5–9), along with fewer pneumonias, fewer tracheobronchitis episodes, fewer recannulations (4 vs 9), and shorter hospital stays (48 vs 62 days). In the final analysis, prolonged capping as a gatekeeper appears unnecessary; a simple, pragmatic criterion focused on secretion burden and 5-minute occlusion, under conditions of cuff deflation and adequate humidification, is more appropriate for patients without swallowing impairment.

R24: For tracheostomized patients weaned from mechanical ventilation, experts suggest coordinated multidisciplinary interventions with team training to accelerate decannulation.

### Expert opinion, strong agreement

Care of tracheostomized ICU patients involves multiple disciplines (intensivists, otolaryngologists, respiratory therapists, physical medicine and rehabilitation, physiotherapists, speech-language pathologists, nurses, dietitians, etc.) and requires specific expertise. Organizing these resources as specialized multidisciplinary teams or care “bundles” has been increasingly reported since the early 2000s. The literature comprises 18 studies: 12 historical before-and-after retrospective cohorts following implementation of multidisciplinary care, two prospective observational cohorts without a control group, one prospective observational cohort with a control group, two retrospective observational cohorts, and one narrative review/meta-analysis. Overall methodological quality is moderate to low.

Regarding time to decannulation, the overall body of evidence favours multidisciplinary care: eight studies report significant reductions [78,[148], [149], [150], [151], [152], [153], [154]], one study is non-significant but trends toward earlier decannulation [155], and seven are non-significant or not directly comparable [144,[156], [157], [158], [159], [160], [161]]. For failed decannulation, only one out of eight studies shows a significant benefit [154]; the remaining seven are non-significant or not comparable [144,149,151,[156], [157], [158],^160^]. For ICU length of stay, three studies have reported significant reductions [78,142,151] and four show no difference [153,156,157,159]. No significant or comparable effects were found for respiratory infections [144,149,156] or mortality [144,162]. In summary, tracheostomy decannulation comprises complex processes; despite the low overall level of evidence, experts suggest promotion of coordinated multidisciplinary involvement and staff training, but further studies are necessary.

## CRediT authorship contribution statement

CM and JD wrote the introduction section. CM, AWT, MR wrote the methodology section. All authors contributed to elaboration of the recommendations and to writing the rationales for the recommendations. All authors provided references. CM and JD drafted the manuscript. All authors read and approved the final manuscript.

## Consent for publication

Not applicable.

## Ethics approval and consent to participate

Not applicable

## Funding

These recommendations were supported by the Intensive Care Physiotherapy Society (Société de Kinésithérapie de Réanimation, SKR) and the 10.13039/501100008800French Intensive Care Society (Société de Réanimation de Langue Française, SRLF).

## Availability of data and material

Not applicable.

## Declaration of competing interes

The authors declare that they have no competing interests.
